# Application of Deconvolution in Path Integral Simulations

**DOI:** 10.1021/acs.jctc.4c00564

**Published:** 2024-10-15

**Authors:** Ádám Madarász, Gergely Laczkó

**Affiliations:** † Research Centre for Natural Sciences, Magyar Tudósok Körútja 2, Budapest H-1117, Hungary; ‡ Hevesy György PhD School of Chemistry, Eötvös Loránd University, P.O. Box 32, Budapest H-1518, Hungary

## Abstract

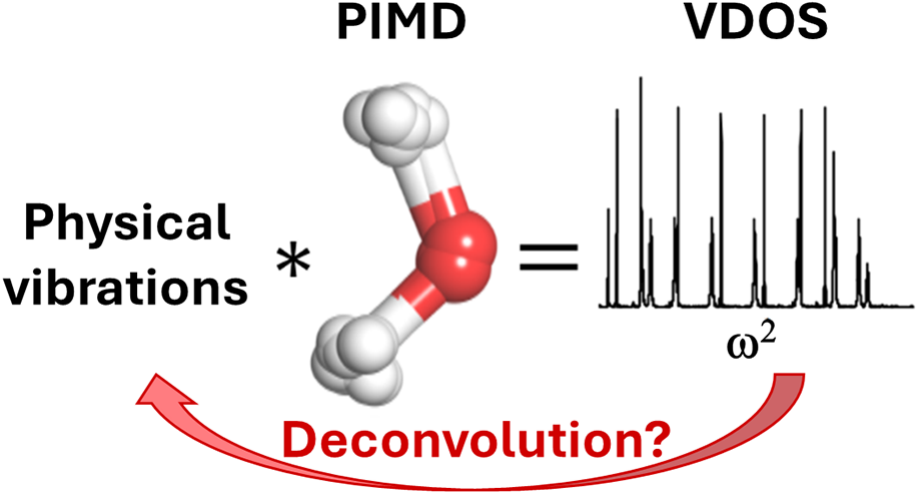

In path integral molecular dynamics (PIMD) simulations, atoms are represented by several replicas connected with harmonic springs, so additional vibrations appear beyond the physical vibrations because of the normal mode frequencies coming from the springs of the ring polymer. In harmonic approximation, the frequencies of these internal modes can be determined exactly from the physical frequencies. We show that this formal effect of the path integral simulations on the vibrations can be considered as a convolution if we use the square of the frequency as an independent variable. This convolution can be represented as a matrix multiplication. The potential of the formalism is demonstrated in two applications. We present an alternative method to determine the power spectrum of thermostats used in PIMD simulations. We also show that in simple anharmonic model systems, the physical frequencies can be obtained from ring polymer molecular dynamics simulations by deconvolution, even in cases where spurious resonances appear.

## 1 Introduction

Nuclear quantum effects (NQEs) in large systems can be investigated with path integral molecular dynamics (PIMD) simulations.^1−3^ In principle, the number of replicas or beads should be infinity to obtain exact results, but in practice the number of replicas is increased until the convergence is reached. In the standard path integral approach which is based on a second order Trotter decomposition of the Boltzmann operator,^4^ there are two techniques for acceleration of this convergence: path integral generalized Langevin equation thermostat (PIGLET) simulations^5,6^ and path integral quantum thermal bath (PIQTB).^7^ These methods are based on the generalized Langevin equation (GLE) thermostat,^8−10^ and the quantum thermal bath (QTB).^11^ Dynamic properties cannot be calculated from standard PIMD simulations. The vibrational density of states (VDOS) computed from PIMD simulations has no physical meaning. Recently, Althorpe reviewed path integral methods for the investigation of dynamic properties.^12^ There are basically two approximate methods for studying dynamic properties: centroid molecular dynamics (CMD) and ring-polymer molecular dynamics (RPMD), but both have problems in the calculation of the vibrational density of states: curvature problem in CMD and spurious frequencies appear in the RPMD spectrum. These problems can be reduced with quasicentroid molecular dynamics (QCMD) and thermostated RPMD (TRPMD). In this work, we present an alternative way to determine the power spectrum of PIMD thermostats. We also demonstrate in simple cases such as OH and H_2_O molecules that physical frequencies can be obtained by deconvolution from RPMD simulations. Despite the relative simplicity of these systems, for a given temperature and number of replicas, spurious frequencies appear in the VDOS. These two molecules show two different cases: the stretching vibration interferes with an internal mode of rotation/libration (OH molecule) or with an internal mode of bending (water molecule).

## 2 Methods

For simulations, we used the CP2K software with version 2023.1.^13^ Default settings were applied unless otherwise noted. We performed 10 ps long PIMD simulations with a time step of 0.25 fs followed by 10 ps long RPMD simulations. For the PIMD simulations the path integral Langevin equation (PILE) thermostat was applied with a time constant of 1000 fs.^14^ Independent trajectories were generated with different seed numbers used for the thermostat. In the RPMD simulations, the thermostat was simply turned off. The RPMD VDOS was calculated from the velocities of the centroids. We checked that the total energy remained constant in the RPMD simulations. For the computation of the VDOS, 10000 independent simulations were generated. To reproduce the spurious frequencies from the literature we used the same replica number, *P* = 16, but we checked the results with a larger replica number as well (*P* = 32). After the equilibration, the translation and the rotation of the whole system was removed, and then 10 ps RPMD simulations were performed. Before we computed the vibrational density of states, the translation and the rotation of each replica was removed. Examples of the input files can be found in the Supporting Information.

### 2.1 Simulation of OH Radical

We performed two types of simulation for the OH molecule. First, we ran simulations in one dimension: the coordinates of the atoms were constrained to one dimension, and the center of mass was also fixed for each bead. The second type of simulation corresponds to the literature when the spurious frequencies can be observed. The atoms could move freely in three dimensions as described above. The simulation temperature was 436.5 K. We used the same parameters for the Morse interaction potential as Rossi et al.^15,16^

### 2.2 Simulation of Water Molecule

The simulation temperature was 380.8 K. We used the same parameters for the water model as Marx and co-workers.^17^

## 3 Theory

### 3.1 Path Integral Molecular Dynamics

We follow the derivation of Ceriotti and Brieuc.^5,7^ Here, we briefly show the corresponding equations. In CP2K,^13^ both the PIGLET and PIQTB methods are implemented, following the RPMD representation.^17^ If the number of beads is *P*, then the simulation temperature is *PT*. The *m* masses are the real physical masses, and the final potential energy is the average of the potential energies of the beads. The Hamiltonian in the PIMD simulations^18^:
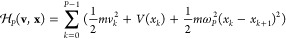
1where **x** = (*x*_0_, *x*_1_, *x*_2_, ···, *x*_*P*–1_) and **v** = (*v*_0_, *v*_1_, *v*_2_, ···, *v*_*P*–1_) are the coordinates and velocities, *x*_*P*_ ≡ *x*_0_, *v*_*P*_ ≡ *v*_0_. *V* is the potential energy. ω_*P*_ is the chain frequency:
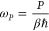
2where β and *ℏ* are the thermodynamic beta and the reduced Planck constant. The potential energy is the average potential energy of the beads:
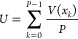
3The classical kinetic energy:
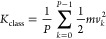
4According to the equipartition theorem, the average classical kinetic energy of the beads:
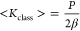
5The spring energy per bead:
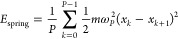
6The primitive estimator of the kinetic energy:
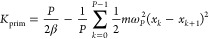
7

### 3.2 PIMD for Harmonic Oscillator

We can derive analytically, what we should obtain from the PIMD simulation of harmonic oscillator and we also know the exact quantum results as well. The potential energy of the harmonic oscillator:
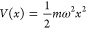
8where ω is the frequency of the oscillator. The average potential energy:
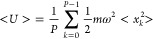
9The Hessian is the second derivative of the Hamiltonian:
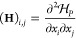
10The diagonal elements are *m*(ω^2^ + 2ω_*P*_^2^) and the off-diagonal elements are – *m*ω_*P*_^2^
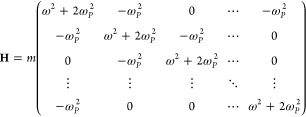
11With the diagonalization of the Hessian, the normal-mode frequencies and vectors can be determined.
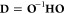
12where **D** is the diagonal matrix and **O** is the matrix with the eigenvectors. Normal mode frequencies can be expressed analytically:
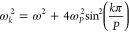
13With normal mode transformation one can obtain the normal coordinates **q** from **x**.
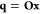
14Since
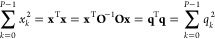
15the mean square fluctuation of the position is
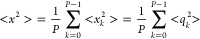
16Based on the equipartition theorem, for each normal mode, the mean energy:
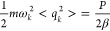
17From this we obtain the mean square fluctuations of the normal coordinates:
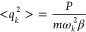
18Combining eqs 16 and 18
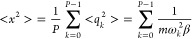
19The average potential energy:
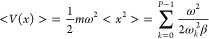
20It can be shown that the potential energy converges to the exact quantum solution^19^ as *P* approaches infinity:
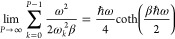
21where coth denotes the hyperbolic cotangent function.

### 3.3 Functional Equation for the Path Integral Weight Functions

We are looking for a *w* (ω) weight function that we can use to modify eq 20 to obtain the exact result for the potential energy.
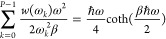
22The final equation is
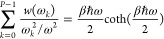
23Equation 23 is a functional equation, that can be easily solved for *P* = 1:
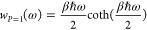
24This weight function is used as a quantum harmonic correction in several places.^8−11,20−22^ The solution of eq 23 is not that straightforward for *P* > 1. Before we show the numerical solution of this functional equation, let us define a reduced angular frequency ω_*T*_ = 2/(β*ℏ*) and use ω*′* = ω/ω_*T*_ for simplicity:
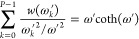
25This way, the expression of the normal-mode frequencies (eq 13) becomes simpler as well:
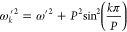
26

### 3.4 Previous Solution of the Functional Equation

Ceriotti used an iteration procedure to solve the functional equation,^5^ and Brieuc implemented it in CP2K.^7^ The initial weight function of the iteration is
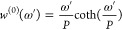
27The next weight function in the iteration is
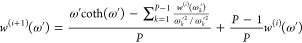
28This solution is fast and gives satisfactory results in path integral simulations, but we wanted more accurate weight functions than what are available from CP2K.

## 4 Results and Discussion

### 4.1 Transformation of the Problem to a Toeplitz System

We developed another procedure for the solution of the functional equation. If eq 25 is divided by ω_*k*_^2^ then ω_*k*_*′* is separated from ω*′*:
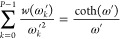
29We can replace the variable of ω*′* by its square, *z*:
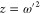
30Let us introduce two new functions:
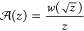
31
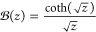
32The functional equation of eq 29 becomes even simpler:
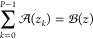
33The *c*_*k*_ differences between the *z*_*k*_ and *z* values are constant, they depend on the number of replicas *P* and the *k* index:
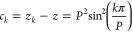
34With a delta function of
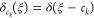
35we can generate the  functions:
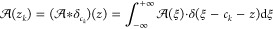
36where ∗ denotes convolution.

If we use the η function, the sum of the dirac delta functions:
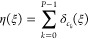
37we obtain the sum of the  functions as
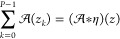
38and we can eliminate the variable *z*_*k*_ from eq 33, and the functional equation can be written as a convolution:
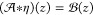
39The convolution above can be represented by a matrix-vector multiplication:
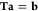
40where **a** and **b** are vectors with *n* elements:
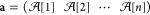
41
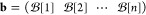
42and **T** is a triangular Toeplitz matrix:
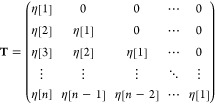
43

### 4.2 Iterative Solution of the Toeplitz System

**Figure 1:**
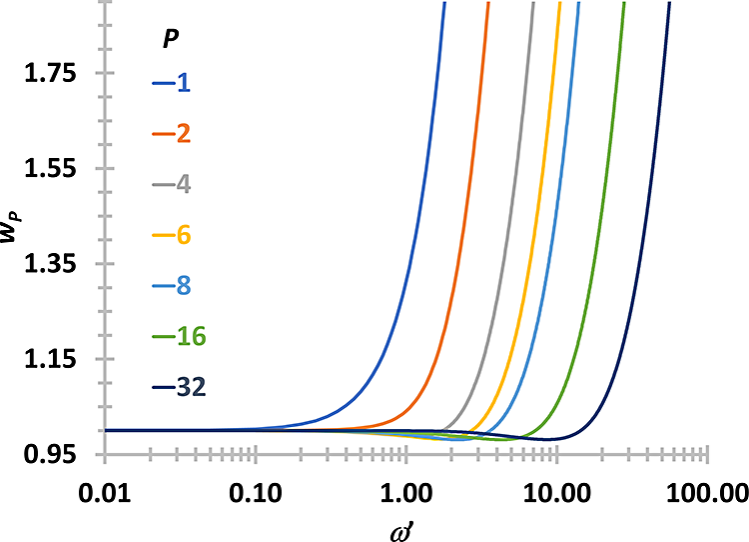
Weight functions for different number of beads (*P*) obtained with the iterative method proposed in this section (after 100 iterations).

The properties and solutions of Toeplitz systems have already been extensively investigated in the past decades.^23^ The “naive” solution of **a** = **T**^–1^**b** often does not work properly.^24^ In our particular case the problem is that if we use only a finite matrix for the representation of **T** then the “naive” solution is not physical i.e. the **a** function can be discontinuous or negative. We used the following iterative equation to solve the functional equation.
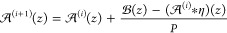
44In matrix representation:
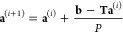
45The first approximation is
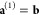
46We determined the functions  with 100 iterations for *P* = 2, 3, 4, 6, 8, 16, and 32. This method is very effective and accurate in the cases where the force constants are equidistant in the η(*z*) functions *P* = 2, 3, 4, and 6. Finally, the weight functions were determined from eq 31:
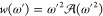
47The weight functions are shown in Figure 1.

Compared to previously determined weight functions that were obtained from CP2K, the relative difference is always less than 0.4%.

**Figure 2:**
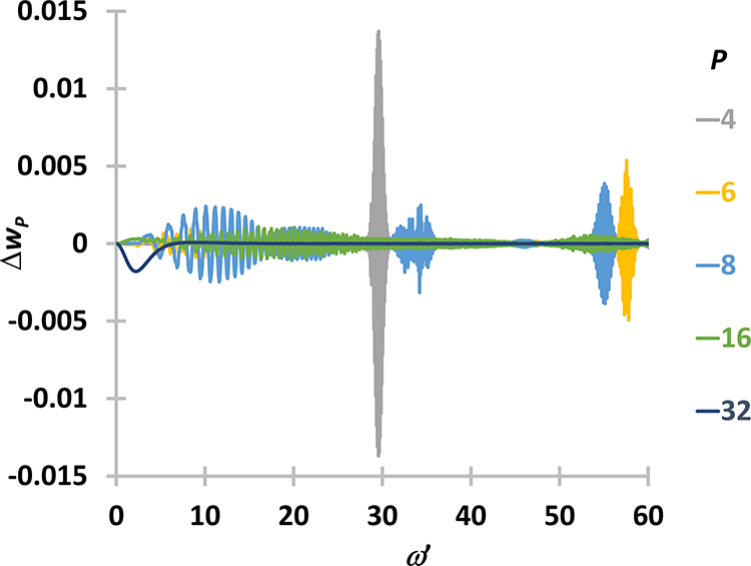
Difference between the weight functions obtained with the method proposed here and the weight functions obtained with the method of Ceriotti et al.^5^ and Brieuc et al.^7^ as implemented in CP2K for various number of beads (*P*).

The differences between the weight functions obtained with the present method and the previous method are shown in Figure 2.

**Figure 3:**
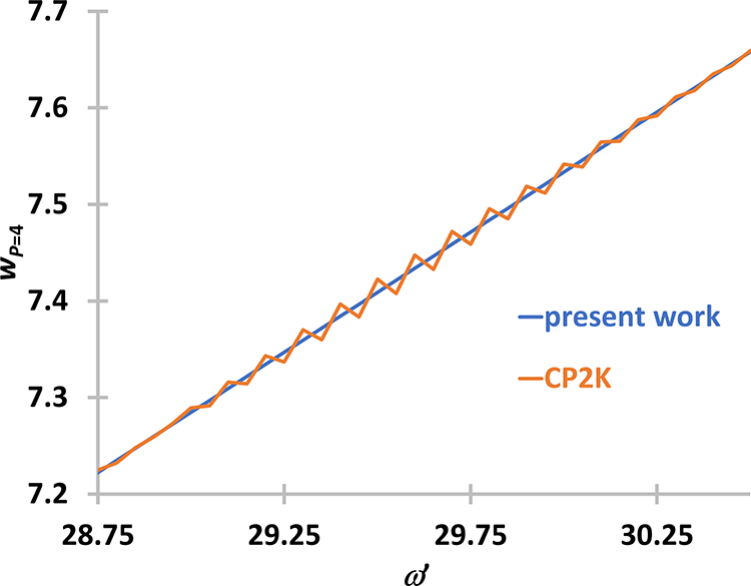
Comparison of the weight functions for *P* = 4. Weight functions were either obtained with the method proposed in this section (present work, blue) or with the method of Ceriotti and Brieuc (orange).

For *P* = 2, the difference is negligible, and thus it is not shown in the figure. An oscillating difference can be observed for 4 ≤ *P* ≤ 16. The difference is systematic around 1 for *P* = 32. The largest difference can be observed for *P* = 4. To compare the different weight functions, we plot the regions where the largest differences occur. The two weight functions are shown in Figure 3 for *P* = 4, and it turns out that the oscillations are present in the CP2K solutions and not in the present results. Obviously, these small oscillations are unphysical. This effect might just be coming from the numerical implementation of the method in CP2K, and is not necessarily an intrinsic problem of the approach developed by Ceriotti. The effect of these small oscillations is negligible in PIGLET or QTB simulations.

**Figure 4:**
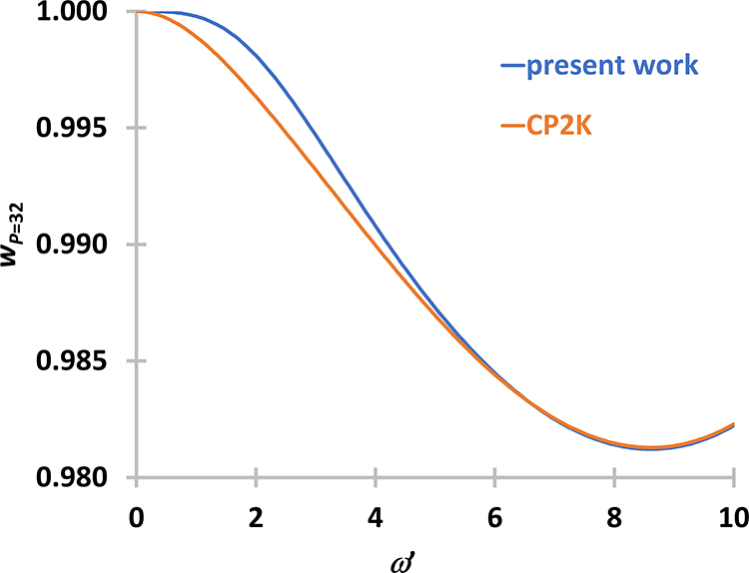
Comparison of the weight functions for *P* = 32. Weight functions were either obtained with the method proposed in this section (present work, blue) or with the method of Ceriotti and Brieuc (orange).

When we compare the weight functions for *P* = 32 there is no oscillation at all, but the present method gives larger values when ω*′* is around 2–3 (see Figure 4). The problem is that 100 iterations is not enough for full convergence for *P* ≥ 32. If we increase the number of iterations, then the weight function starts to diverge because of numerical errors.

**Figure 5:**
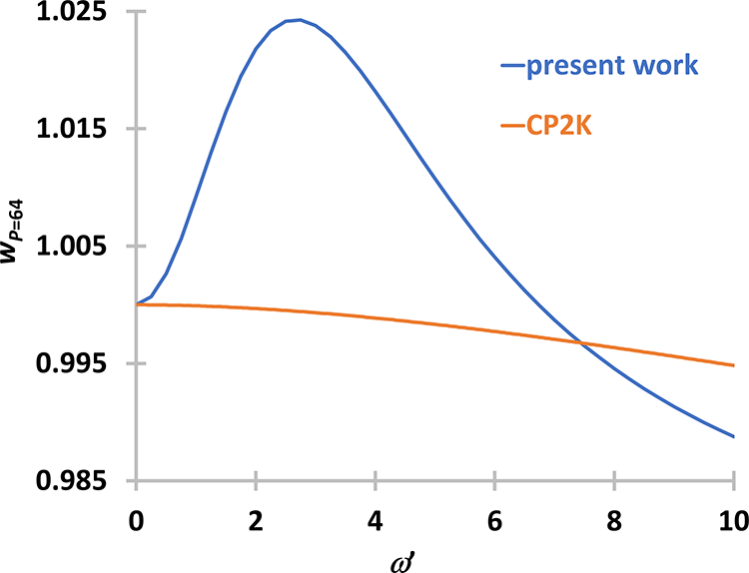
Comparison of the weight functions for *P* = 64. Weight functions were either obtained with the method proposed in this section (present work, blue) or with the method of Ceriotti and Brieuc (orange).

The sign problem appears in the summation of numbers with an alternating sign. The problem arises when the exact sum is smaller than the last digits of the largest numbers in the series. We used quadrupole precision for the calculations of the weight functions, but this was not enough for *P* = 64. In Figure 5, it can be seen that the present approach fails, giving a local maximum around 3 and similar curves were not observed for smaller *P*-s.

Although our method is effective for small *P*, it becomes ineffective for *P* = 32 and fails for *P* ≥ 64. It is quite possible that there are more effective algorithms in the literature of Toeplitz systems, but we have not investigated them because the current solutions are accurate enough for the application of PIMD simulations.

In the GLE thermostats and in QTB, there are different versions, implementations. It is possible to apply thermostats only to the internal, noncentroid vibrations. In that case, the functional equation changes and the centroid mode (*k* = 0) is separated in eq 25:
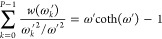
48In this case, the previous procedure can also be performed, only the function  needs to be modified.

### 4.3 Determination of the Vibrational Density of States

Now we show that the application of the variable *z* and the function η can be very useful in the calculation of the vibrational density of states (VDOS or *C*_*vv*_). For the determination of the quantum-corrected thermodynamic properties, the velocity autocorrelation functions (VACF or *c*_*vv*_) are computed from molecular dynamics simulations that can be defined as follows:
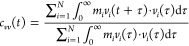
49where *m*_*i*_ and *v*_*i*_ are the mass and the velocity of the *i*th atom as a function of time (*t*). With this definition, the autocorrelation function is always 1 at zero time, i.e., *c*_*vv*_(0) = 1. The vibrational density of states is the Fourier transform of the autocorrelation function:
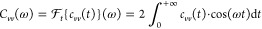
50In the first step, we replace the ω variable with *z* preserving the area under the curve:
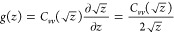
51We can determine the *c*_*vv*_, *C*_*vv*_ and *g* functions at classical (cl), path integral (PI), and quantum (q) levels. Assuming independent harmonic oscillators, we can use the following relationships between the different theoretical descriptions. The vibrational density of state at the quantum level can be determined simply from the classical *C*_*vv*_:
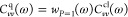
52The relationship between the classical and path integral models is not that simple using the variable ω, but with *z* it becomes a linear transformation:
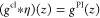
53The latter equation in matrix representation:
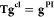
54where the vectors are
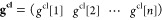
55
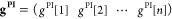
56

Now, eq 53 can be written as a discrete convolution:
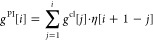
57

Using the equations above, one can calculate the vibrational density of states *C*_*vv*_^PI^(ω) from PIMD simulations, and it can be transformed to an approximate *C*_*vv*_^q^(ω). If we assume independent harmonic oscillators, then we can reconstruct the real, quantum vibrational density of states, *C*_*vv*_^q^(ω). The steps are summarized here:

1. Generation of PIMD trajectories.
2. Calculation of the path integral vibrational density of states *C*_*vv*_^PI^(ω).
3. Change in the variable from ω to *z* = ω_*k*_*′*^2^ to get *g*^PI^(*z*).
4. Deconvolution of *g*^PI^(*z*) to obtain *g*^dec^(*z*).
5. Change in variable from *z* back to ω to get *C*_*vv*_^dec^(ω).
6. Multiplication of *C*_*vv*_^dec^(ω) with the weight function *w*_*P*=1_(ω) to obtain an approximation for the quantum vibrational density of states *C*_*vv*_^q^(ω).

In harmonic systems *g*^dec^(*z*) and *g*^cl^(*z*) should be identical. In anharmonic cases, they can be different, since the frequencies can be shifted.

The most problematic step is deconvolution, that is, solving the Toeplitz equation to obtain *g*^dec^(*z*). Again, the “naive” solution can be unphysical and unstable because of the noise. To obtain a realistic and stable solution, we need to perform a nonnegative or a bounded-variable least-squares (NNLS or BVLS) optimization,^25,26^ i.e. minimizing the next function
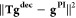
58subject to
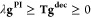
59where λ is an arbitrary scaling factor to avoid overfitting of the shape and position of the peaks. More details about this λ parameter can be found in the Supporting Information. Since interference between internal and physical vibrations can make deconvolution difficult, the objective function of eq 58 and the conditions in eq 59 were evaluated outside the range of physical frequencies, that is, 10000 cm^–1^ > ω > 5000 cm^–1^. We had to use the upper limit because of the broadening of the peaks due to the finite simulation time. Furthermore, we assume that the function *g*^dec^(*z*) is zero above the physical frequencies ω > 5000 cm^–1^.

After we have found the *g*^dec^ function, we just replace the *z* variable
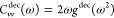
60and multiply with the weight function.
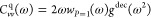
61

*C*_*vv*_^dec^(ω) and *C*_*vv*_^q^(ω) contain the same information. *C*_*vv*_^dec^(ω) and *C*_*vv*_^cl^(ω) will be compared below along with the vibrational density of states computed from the centroid velocities *C*_*vv*_^RPMD^(ω).

**Figure 6:**
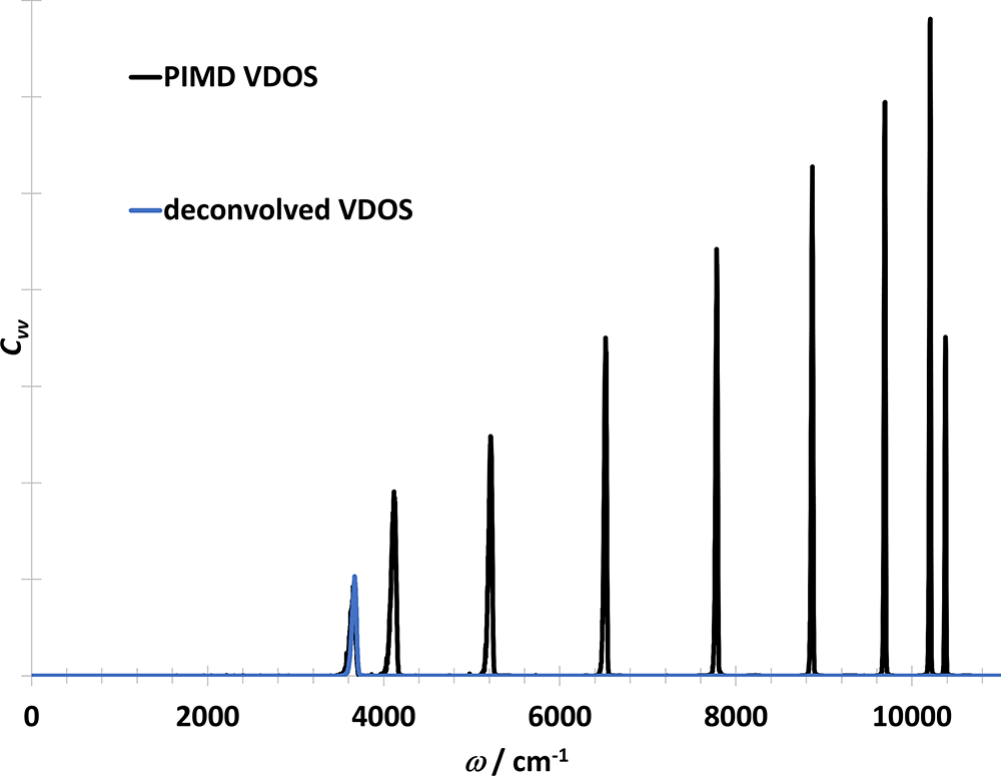
Vibrational density of states of OH radical in one dimension obtained from the PIMD trajectory directly (i.e., including the ring polymer normal modes) (black) and after deconvolution (blue).

**Figure 7:**
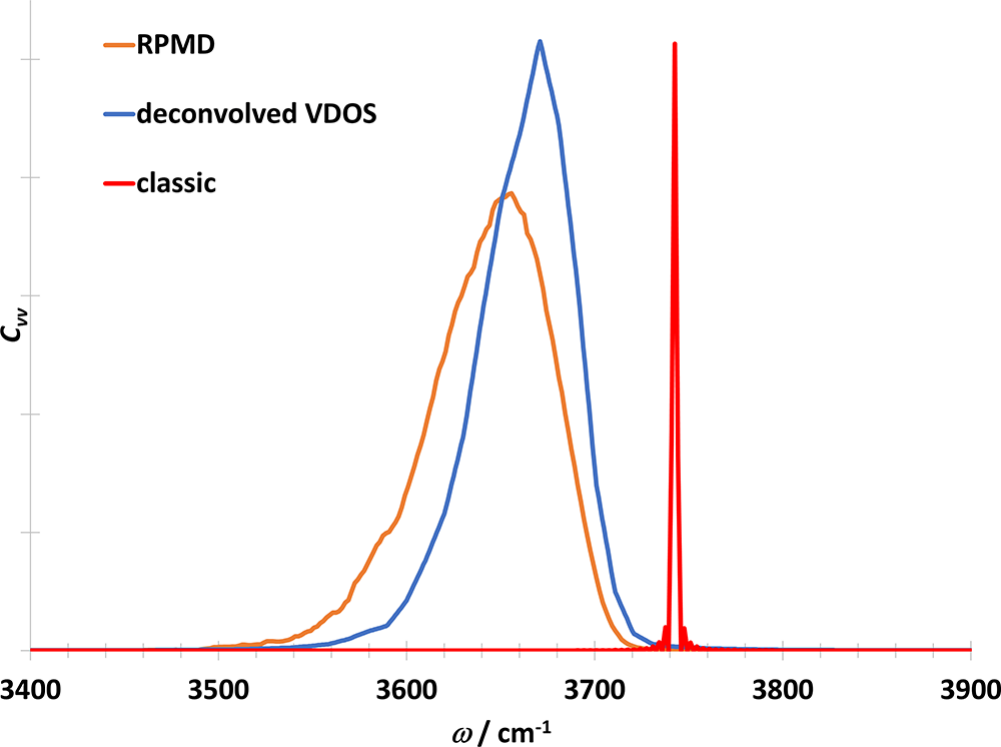
Vibrational density of states of OH radical in one dimension obtained with various methods. Classical spectrum is scaled down by a factor of 20 for better visualization.

To test the feasibility of our approach, we first investigated an OH radical which represents a one-dimensional test case.^15,17^ We performed RPMD and classical simulations using the Morse potential. Since all translational and rotational modes were eliminated in the simulations, no spurious frequencies were observed in the spectrum (see Figure 6). The RPMD and the deconvolved VDOS are shown in Figure 7 compared to the VDOS from the classic simulation. Both peaks are red-shifted compared to the classical peak. The shape and the position of the two peaks are similar; the deconvolved peak is slightly blueshifted compared to the RPMD peak. This small, 15 cm^–1^ difference is due to the fact that during the deconvolution, several peaks are mixing together, and the distances between these peaks can deviate slightly from what we would expect from eq 13 because of the anharmonicity.

**Figure 8:**
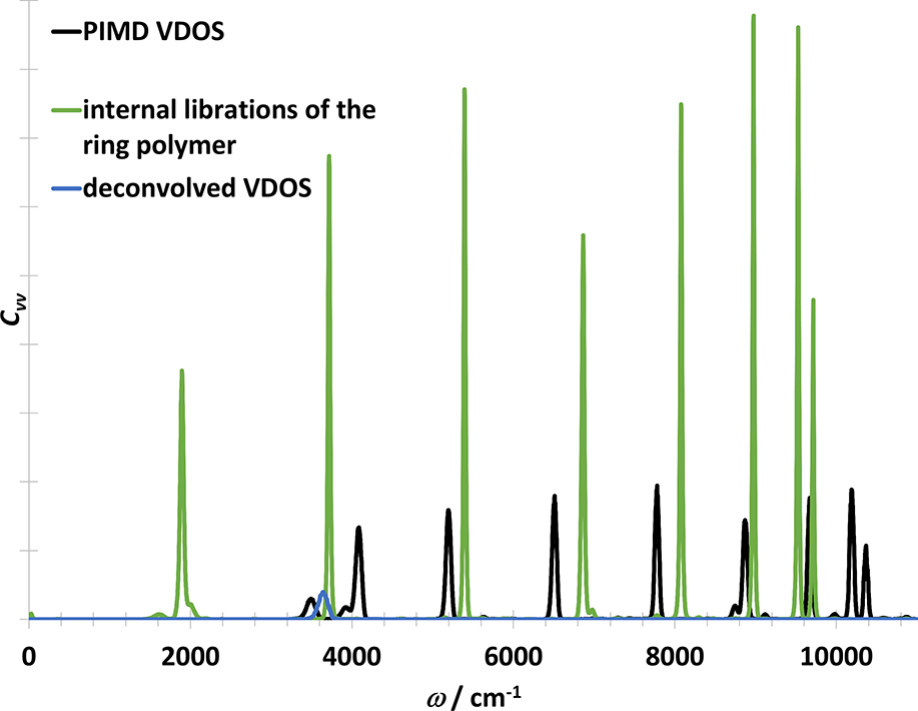
Vibrational density of states of OH radical in three dimensions obtained with various methods from the PIMD trajectory directly (i.e., including the ring polymer normal modes). Black curve contains the vibrational part. Internal librations of the ring polymer is shown with green. Blue curve was obtained from the deconvolution of the black curve.

**Figure 9:**
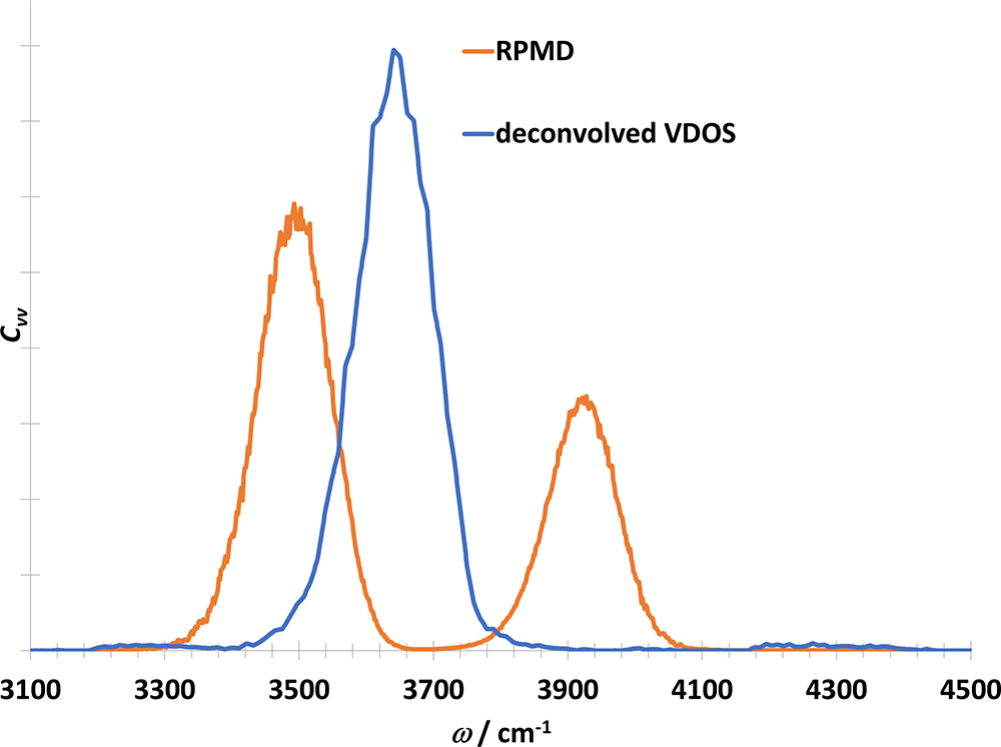
Vibrational density of states of OH radical in three dimensions from RPMD simulation (orange) and by the deconvolution of the VDOS (blue).

The next model case, the OH molecule in three dimensions, is taken from refs (15,17). In contrast to the one-dimensional case, an internal mode of libration interferes with the physical vibration (see Figure 8). This causes an artificial splitting of the peak in the RPMD spectrum (see Figure 9). The deconvolved VDOS contains only one peak. Compared to the TRPMD spectrum from ref (15), the deconvolved VDOS spectrum is narrower, but blueshifted with 36 cm^–1^. Using a larger number of beads (*P* = 32), the peak is slightly narrower (see Figure S3 in the Supporting Information).

**Figure 10:**
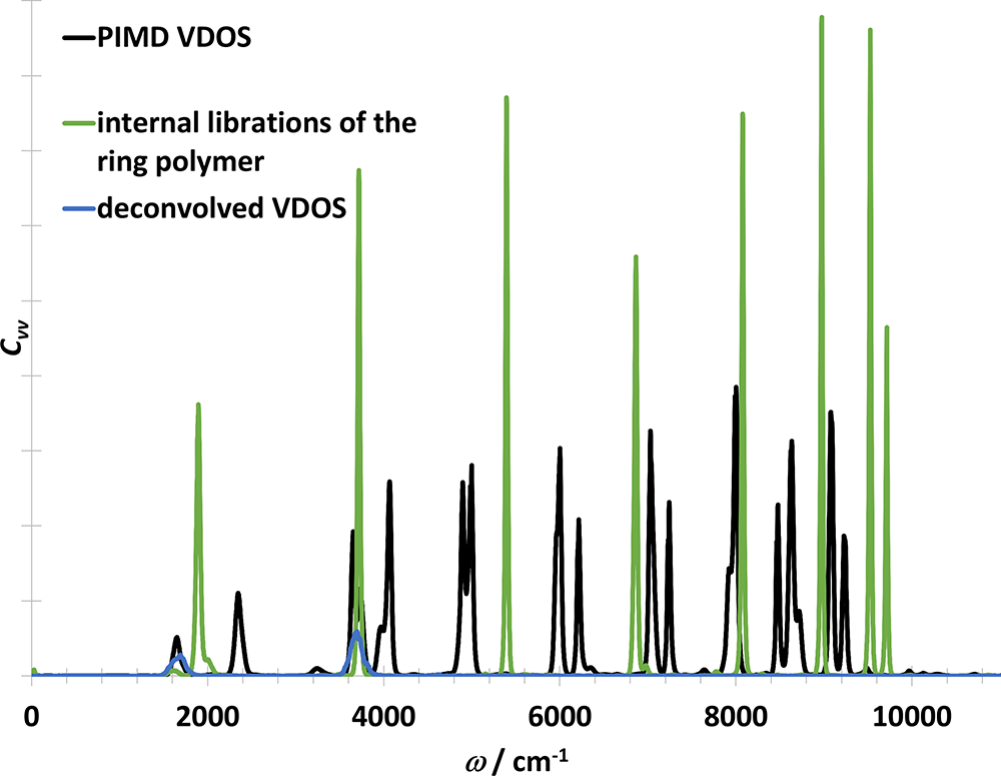
Vibrational density of states of water molecule obtained with various methods from the PIMD trajectory directly (i.e., including the ring polymer normal modes). Black curve contains the vibrational part. Internal librations of the ring polymer is shown with green. Blue curve was obtained from the deconvolution of the black curve.

**Figure 11:**
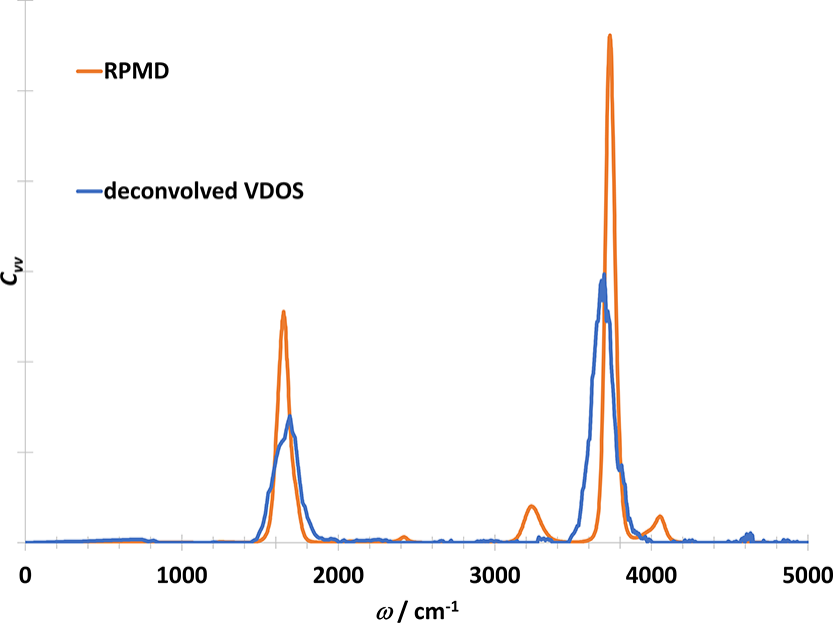
Physically meaningful vibrational density of states of water molecule from RPMD simulation (orange) and by the deconvolution of the VDOS (blue).

To further test the applicability of our approach, we investigated a single harmonic water model, where the resonance problem occurs in RPMD simulations between the real physical vibrations and the internal modes. In Figure 10, it can be seen that after deconvolution 2 peaks remain that belong to the real physical vibrations. The deconvolved spectrum is compared to the RPMD spectrum in Figure 11. There are two small peaks around 3200 and 4100 cm^–1^ in the RPMD spectrum. These are the spurious frequencies that were identified by Marx et al. and it is because of the resonance of the stretching and one of the internal modes of the bending vibration.^17^ These spurious frequencies are not present in the deconvolved spectra.

**Figure 12:**
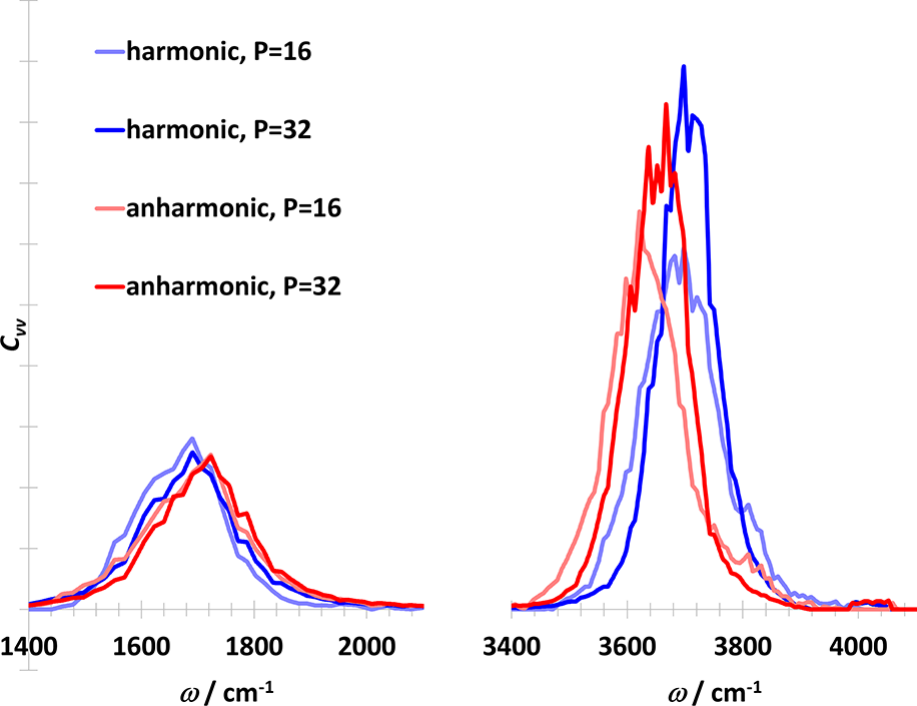
Deconvolved vibrational density of states with different water models and replica numbers.

The physical frequencies are reproduced well, with only a small amount of noise left. We checked the VDOS functions with a larger number of beads (*P* = 32), as well as anharmonic water model. The spectra are shown in Figure 12. For all cases the peaks of the bending mode are very similar. For the anharmonic water model, the peak of OH stretching is redshifted with 50 cm^–1^ compared to the peak of the harmonic water model. The OH stretching peaks are slightly narrower with *P* = 32 than with *P* = 16, which means that the spectrum is not yet strictly converged with *P* = 32.

These results can be considered only as a proof-of-concept, and this approach should be thoroughly tested. Can this approach handle vibrations related to translation and rotation? How well is it working at the low frequency region? How fast is the convergence of the spectrum with the number of replicas? These questions can be answered in future work.

## 5 Conclusions an Perspectives

The main result of our work is that we showed that the formal effect of path integral simulations on the frequencies can be considered as a convolution if we use the square of the frequency as an independent variable. The convolution can be represented as a matrix multiplication in eqs 40 and 54. These two examples show the utility of this approach. We proposed a new method for calculating weight functions that can be used in the development of new GLE thermostats or alternatively to extend Generalized Smoothed Trajectory Analysis (GSTA)^22^ to path integral simulations.

Generally, time-dependent/dynamic properties cannot be determined from standard PIMD simulations. Here, we showed an alternative way to obtain physical frequencies from RPMD simulations. Although the TRPMD method successfully eliminates spurious frequencies, it artificially broadens the peaks.^15^ In TRPMD the internal vibrational modes are thermostated, to decouple them from the physical vibrations. Our deconvolution approach is a postprocessing method for RPMD simulations that facilitates the identification of unphysical frequencies. Further comparative studies between the two methods are required to assess their performance and reliability across different systems.

Only a few attempts are made in the literature to reconstruct the physical VDOS by deconvolution. Rossi deconvolved the effect of thermostat in TRPMD simulations.^27^ Later, Kapil extended that approach to PIGLET simulations.^28^ Beyond these studies, the present approach may be applicable in the future to standard PIMD simulations.

## Data Availability

Weight functions: https://zenodo.org/records/10702413; Wolfram code to solve the path integral weight function problem: https://www.wolframcloud.com/obj/d161accb-b8eb-4f23-8964-fa9ac830c7e1; Fortran code to solve the path integral weight function problem: https://github.com/madaraszadam/PI_weight_functions. Wolfram code for the deconvolution of the path integral VDOS: https://www.wolframcloud.com/obj/eba0363a-dd44-41b9-a430-936c5c31a80e.

## Supporting Information Available

Analysis of the *λ* scaling factor (PDF)
Input files of water and OH (ZIP)
