# Supplementary material for: Application of Deconvolution in Path Integral Simulations
Source: J Chem Theory Comput. 2024 Oct 15;20(21):9562–70. doi: 10.1021/acs.jctc.4c00564 (PMC11562373; doi:10.1021/acs.jctc.4c00564)
Supplement: Supplementary file 1 — ct4c00564_si_001.pdf [file ct4c00564_si_001.pdf]

Supporting Information: Application of Deconvolution in Path Integral Simulations

Ádám Madarász*,† and Gergely Laczkó†,‡

†*Research Centre for Natural Sciences, Magyar Tudósok Körútja 2, H-1117 Budapest, Hungary*

‡*Hevesy György PhD School of Chemistry, Eötvös Loránd University, P.O. Box 32, Budapest, H-1518, Hungary*

E-mail: madarasz.adam@ttk.hu

In **Figure S1-2**, we show the effect of the λ parameter in the deconvolution. $\lambda = 1$ and $\lambda = \infty$ represent two extreme cases. $\lambda = \infty$ mmeans there is no upper limit for the function. In this case, the VDOS function is overfitted, and several small peaks appear in the spectrum. The problem is that the areas under the vibrational modes should be the same according to the equipartition theorem, but these deviations are fitted at the price that small peaks appear at higher frequencies. Basically, the maximum of these peaks can be regularized with the lambda parameter. $\lambda = 1$ is a too strict condition. The physical peaks are reduced too much because of the deviation in the peak sizes and positions. In these particular systems, $\lambda = 5$ is a good compromise between the two extreme cases, representing a good signal-to-noise ratio.

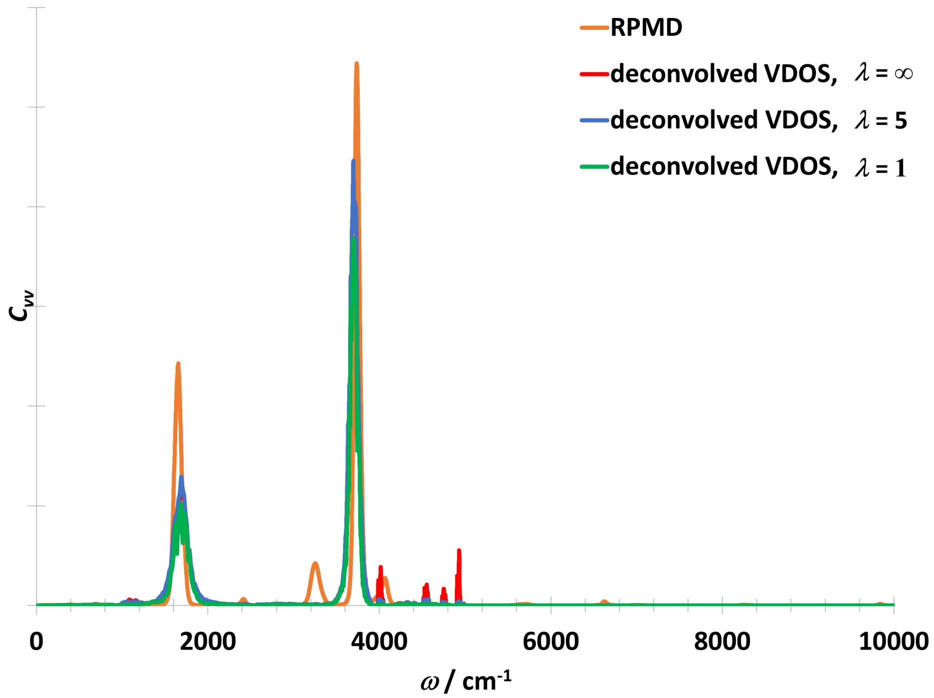

Figure S1. Vibrational density of states of water molecule. $P = 32$, $T = 380.8$K

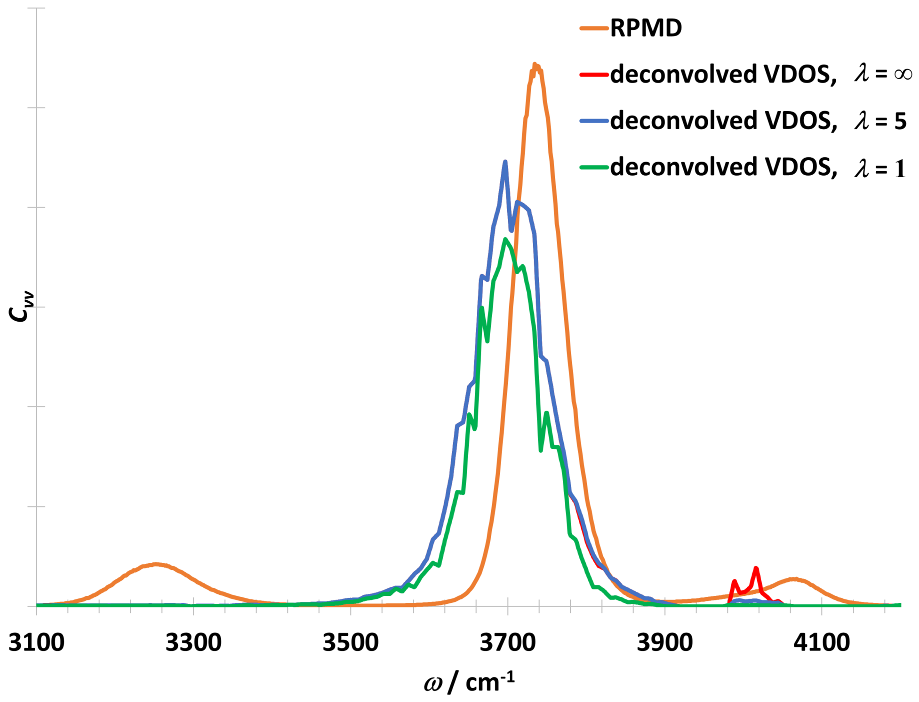

Figure S2. Vibrational density of states of water molecule, zoomed. $P = 32$, $T = 380.8$K

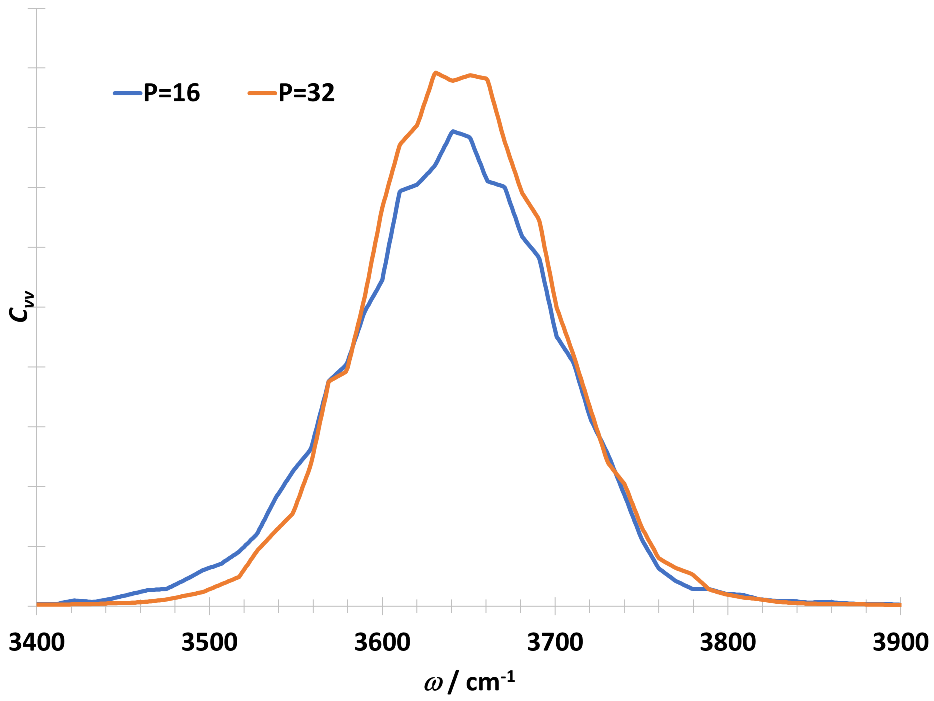

Figure S3. Deconvolved VDOS of OH radical in three dimensions with different replica

numbers. $T = 436.5\text{K}$
